# Identification of systemic immune response markers through metabolomic profiling of plasma from calves given an intra-nasally delivered respiratory vaccine

**DOI:** 10.1186/s13567-014-0138-z

**Published:** 2015-02-14

**Authors:** Darren W Gray, Michael D Welsh, Simon Doherty, Fawad Mansoor, Olivier P Chevallier, Christopher T Elliott, Mark H Mooney

**Affiliations:** Institute for Global Food Security (IGFS), School of Biological Sciences, Queen’s University Belfast (QUB), Belfast, Northern Ireland BT9 5AG UK; Veterinary Sciences Division (VSD), Agri-Food and Biosciences Institute (AFBI), Belfast, Northern Ireland BT4 3SD UK

## Abstract

Vaccination procedures within the cattle industry are important disease control tools to minimize economic and welfare burdens associated with respiratory pathogens. However, new vaccine, antigen and carrier technologies are required to combat emerging viral strains and enhance the efficacy of respiratory vaccines, particularly at the point of pathogen entry. New technologies, specifically metabolomic profiling, could be applied to identify metabolite immune-correlates representative of immune protection following vaccination aiding in the design and screening of vaccine candidates. This study for the first time demonstrates the ability of untargeted UPLC-MS metabolomic profiling to identify metabolite immune correlates characteristic of immune responses following mucosal vaccination in calves. Male Holstein Friesian calves were vaccinated with Pfizer Rispoval® PI3 + RSV intranasal vaccine and metabolomic profiling of post-vaccination plasma revealed 12 metabolites whose peak intensities differed significantly from controls. Plasma levels of glycocholic acid, N-[(3α,5β,12α)-3,12-Dihydroxy-7,24-dioxocholan-24-yl]glycine, uric acid and biliverdin were found to be significantly elevated in vaccinated animals following secondary vaccine administration, whereas hippuric acid significantly decreased. In contrast, significant upregulation of taurodeoxycholic acid and propionylcarnitine levels were confined to primary vaccine administration. Assessment of such metabolite markers may provide greater information on the immune pathways stimulated from vaccine formulations and benchmarking early metabolomic responses to highly immunogenic vaccine formulations could provide a means for rapidly assessing new vaccine formulations. Furthermore, the identification of metabolic systemic immune response markers which relate to specific cell signaling pathways of the immune system could allow for targeted vaccine design to stimulate key pathways which can be assessed at the metabolic level.

**Electronic supplementary material:**

The online version of this article (doi:10.1186/s13567-014-0138-z) contains supplementary material, which is available to authorized users.

## Introduction

The vaccination of farm animals against endemic, genetically evolving and emerging pathogens is important not only to ensure animal health, but also to reduce the costs associated with disease losses, either clinical or subclinical. Successful vaccination leads to the production of specific T and B cell effector immune responses that assist in the control of infection within the animal. This results in the generation of virus neutralizing antibodies that recognize the pathogen, specific effector T-cell responses and the development of an immune memory response helping to protect against future exposure to the infection. Continuous vaccine development is required to address virus evolution, new and emerging viral threats and to improve vaccine efficacy against currently managed pathogens. However, assessment of new vaccine candidates, adjuvant and novel vaccine carrier systems (such as nanoparticles) using animal trials is extremely expensive and can take several months to years (investigation of short term immune responses, vaccine-challenge studies and field trials and long term immune protection against natural wildtype virus challenge) [1]. Although the expenditure associated with animal vaccine development is not fully disclosed, the estimated budget required to develop a single FDA approved vaccine for human studies is estimated in the region of $1-2 billion [2]. The majority of these costs are attributed to the high failure rate of vaccine candidates/formulations, with only 1 in every 10 000 vaccine formulations gaining approval by the FDA [3]. New vaccine candidates may initially be assessed in mice models, however this does not necessarily translate to performance in the target species and animal trials can have poor efficacy [4] or fail to induce immune protection at all [5] as a result of species differences in immune systems [3]. Furthermore, many factors such: as immune system maturity, vaccine delivery route, concurrent infections, poor nutrition and the presence of maternally derived antibodies can affect vaccine efficacy [6]. As the expenditure associated with candidate vaccine development escalates with clinical trial progression, rapid vaccine screening methods which can assess candidate vaccine effectiveness at early trial stages in vivo are required to minimize financial outlay and improve the speed of vaccine development pipelines.

In the agricultural industry the Bovine Respiratory Disease (BRD) complex is considered to be one of the most significant causes of economic loss in intensively reared cattle worldwide. The associated losses accruing from the elevated mortality and poor growth performance of infected animals [7] coupled with the need for costly therapeutic interventions have a significant negative impact on farm incomes. This disease complex is estimated to result in an annual total economic loss to the US agri-food industry of over $2 billion, with treatment and preventative costs approaching $3 billion [8]. Whilst vaccination against the infectious agents involved in BRD pathogenesis is currently employed to manage the disease [9,10], it has not significantly reduced BRD prevalence or severity. Furthermore, it has been observed that some animals fail to develop immune protection despite vaccine treatment, becoming infected with each new seasonal BRD outbreak [11]. Therefore, the development of new methods for screening BRD pathogen vaccine candidates, along with the clear understanding of the host immune response, would provide significant economic and animal welfare benefits to the agricultural industry by speeding up the vaccine development.

Current methods for determining viral vaccine effectiveness in mammalian species include assessment of neutralizing antibody titre [12,13], PCR-based detection of viral load in infected tissues at post-immunization challenge [12], expression of pro- and anti-inflammatory cytokines [13], duration of viral shedding after post-immunization challenge [14], and mortality/morbidity findings at post-mortem [15]. However, the quantitative analysis of pro- and anti-inflammatory cytokines is generally considered to be one of the important indicators of vaccine efficacy, but typically only occurs after preliminary candidate antigen or formulation screening [6], and may require a number of vaccines and challenge studies to fully elucidate the important factors associated with a protective immune response. A greater understanding of not only the proteomic responses to vaccination [16,17], but also the changes at the metabolic level in bio-fluids, such as plasma or serum, may help develop early marker-based screening tests. These can be used to assess the potential of new vaccine candidates and also may provide additional information to improve the rationale behind future vaccine approaches taking into account vaccine delivery routes, vaccine carrier technologies and animal genetics.

Untargeted metabolomic profiling refers to the top down global analysis of small molecules or “metabolites” with molecular weights less than 1 kDa and is typically performed by Nuclear Magnetic Resonance (NMR) or mass spectrometry (MS) techniques, with the later offering greater sensitivity. Ultra Performance Liquid Chromatography (UPLC) coupled to a highly accurate mass spectrometer allows for the separation and determination of elemental composition (and hence the identification) of 1000s of metabolites in a single sample. As metabolites are often end-stage products of biological processes, their analysis can provide a greater understanding of the physiological state of an organism at the time of sampling compared to genomic or proteomic analysis. The application of metabolomic techniques to the analysis of bio-fluids has been found to offer interesting new insights to the understanding of the physiological processes involved in disease development and diagnosis [18-21]. However, to date little emphasis has been placed on assessing the in vivo metabolomic changes induced by immunization procedures and foreign antigen exposure.

This study for the first time reports the metabolomic responses in plasma of calves vaccinated with an intranasal vaccine (Pfizer Rispoval® PI3 + RSV) composed of the modified live viruses Bovine Respiratory Syncytial Virus (BRSV) and Bovine Parainfluenza Virus-3 (BPI3V). BRSV and BPI3V are two of the major viral pathogens involved in the Bovine Respiratory Disease (BRD) complex [7]. The aims of the current study were to identify plasma metabolomic markers of the primary and secondary immune response in calves to administration of a commercial intranasal vaccine containing live modified virus. Importantly, it is well known that vaccination via the parenteral route leads to poor protection at mucosal surfaces. Therefore the studies undertaken here allow vaccine immunology and biomarkers to be identified through the natural route of infection for these respiratory viruses in order to identify the most suitable host defense mechanisms. This is the first untargeted UPLC-MS based metabolomics study to reveal metabolite markers of the immune response occurring during vaccination which may have potential use in the early screening of candidate vaccine effectiveness and aid new vaccine design by increasing the understanding of the processes involved in immune response development post vaccination.

## Materials and methods

### Chemicals and reagents

HPLC grade acetone was purchased from Sigma Aldrich (Dorset, UK). LC-MS grade acetonitrile, water, methanol and chloroform were purchased from Fisher Scientific (Loughborough, UK). All analytical standards for metabolite confirmation were purchased from Sigma Aldrich.

### Experimental design and sample collection

**Figure 1:**
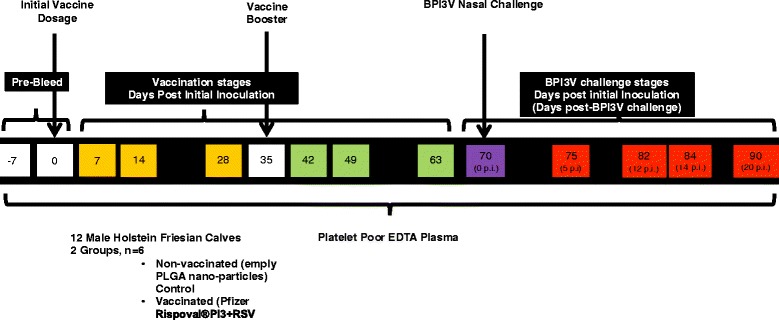
**Sampling points for metabolomic profiling of plasma in calves following vaccination and subsequent BPI3V challenge.** Male Holstein Friesian calves were dosed with Pfizer Rispoval®PI3 + RSV intranasal vaccine (vaccinated) or empty PLGA nanoparticles (non-vaccinated) at day 0. A secondary booster dosage was administered at day 35. At day 70 all animals were experimentally challenged with BPI3V via nasal inoculation. At day 6 during the BPI3V challenge study 3 calves per group were sacrificed for viral isolation and histology. Sampling points for metabolomic profiling of plasma are indicated throughout.

All animal studies were carried out in accordance with the UK Animals (Scientific Procedures) Act 1986 and with the approval of the AFBI Ethical Review Committee. Figure 1 illustrates the experimental design and sample collection days throughout the study. Twelve male Holstein Friesian calves aged between 20 and 25 weeks were divided into two study groups (*n* = 6) and assigned as non-vaccinated or vaccinated calves. Vaccinated calves were treated with Pfizer Rispoval® PI3 + RSV intranasal vaccine (designated vaccinated animals) as per manufacturers instructions, and non-vaccinated calves were treated with empty poly(lactic-co-glycolic) acid PLGA nanoparticles (designated non-vaccinated) prepared using standard double emulsification solvent evaporation technique (w/o/w) [22]. Calves were dosed at day 0 and day 35, and screened weekly for the presence of BPI3V IgG in blood serum using Svanovir-PI3V-Ab kit (Boehringer Ingelheim Svanovir, Uppsala, Sweden) as per manufacturer’s instructions. Calves were sampled at days 0 (pre-dosage), 7, 14, 28 (initial vaccine dosage), 42, 49 and 63 (booster dosage). One week after completion of the vaccination study (day 70) all calves were experimentally challenged with BPI3V through nasal inoculation with 2 mL of virus suspension (TCID_50_ of 10^6.78^/mL) per nostril. Calves were sampled at days 70, 75, 82, 84 and 90 (days 0, 5, 12, 14 and 20 post-BPI3V challenge respectively). Serum was prepared from blood drawn by jugular venepuncture into 10 mL BD Vacutainer® glass serum tubes. The blood was allowed to clot at room temperature for 30 min prior to centrifugation at 2000 *g* to remove the clot. Samples were stored at 4 °C prior to analysis. Blood haematology was monitored weekly during vaccination stages and at days 70, 75, 82, 84 and 90 (post-BPI3V challenge stages). Rectal temperatures were taken at days 14, 28, 42 and 63. Blood was sampled from the calves by jugular venepuncture into 6 ml plastic K3 EDTA Vacuette tubes (Greiner bio-one, Stroudwater, UK) and processed to platelet poor plasma by the double centrifugation method previously described [23]. All samples were processed at random within 2 h of initial blood sampling and stored at −80 °C prior to use.

### Sample preparation

600 μL of plasma was added to 2.4 mL of ice cold acetone, vortexed for 30 s and placed on ice for 15 min. The sample was then deproteinated by centrifugation at 4000 *g* at 4 °C for 20 min. 2.4 mL of supernatant was removed and dried under nitrogen for 45 min at 40 °C using TurboVap LV (Caliper Life Sciences, Hopkinton, USA). Resulting residue was reconstituted in 500 μL of Ultra Pure H_2_O and liquid/liquid extraction of lipids performed by addition of 500 μL of ice-cold Methanol:Chloroform (1:1 v/v) and vortexing for 30 s followed by centrifugation at 16 000 *g* at 4 °C for 15 min. liquid/liquid extraction was repeated and after centrifugation 900 μL of the aqueous layer was removed and dried under nitrogen using TurboVap at 40 °C for 45 min. The residue was reconstituted in 300 μL Ultra Pure H_2_O and filtered by centrifugation at 10 000 *g* at 4 °C using 0.22 μm Constar Spin-X® Centrifuge Tube Filter for 5 min.

### UPLC-MS analysis of Bovine Plasma

UPLC-MS analysis was performed using an Acquity UPLC system coupled to a XEVO G2 Q-TOF (Waters Corporation, Milford, MA, USA. 8 μL of prepared sample extracts were injected onto an Acquity UPLC HSS-T3 C18 column (100 mm × 2.1 mm i.d., 1.8 μm; Waters Corporation, Milford, MA, USA). Column and autosampler temperature were maintained at 50 °C and 10 °C respectively. Chromatographic separation was carried out at a flow rate of 600 μL/min with mobile phase consisting of 99.9% H_2_O/0.1% Formic Acid (A) and 99.9% Acetonitrile/0.1% Formic Acid (B). The elution gradient was as follows: 0–2 min isocratic at 1% of B, 2–14.5 min linear gradient from 1-100% of B, 14.5-17 min isocratic at 100% of B, 17–17.5 min linear gradient from 100-1% of B and finally 17.5-20 min isocratic at 1% of B. Mass spectrometry was performed using a Waters XEVO G2 QTOF operating in positive-ion mode (ESI+) with the capillary voltage set to 1500 V and the sampling cone voltage 30 V. The desolvation and cone gas flows were set at 750 L/h and 100 L/h respectively. Source and desolvation temperatures were 120 °C and 400 °C respectively. Leucine Enkephalin ([M +H]^+^= 278.1141 Da, and [M+ H] = 556.2771 Da) was used for accurate lockmass calibration during data acquisition. Lockmass acquisition settings were: 0.5 s scan time, 30 s interval, 3 scan average, mass window +/− 0.5 Da. Collision energy was only applied on function 2, with ramping between 15 V and 30 V and centroid data was acquired using positive-ion, resolution mode.

### Mass spectrometry data processing

Total Ion Count (TIC) chromatograms and spectra were acquired with MassLynx version 4.1 (Waters) in centroid format and metabolite data was processed using MarkerLynx software (Waters Corporation, Milford, MA, USA). The MarkerLynx method for data extraction and de-convolution was as follows: Ions were extracted from function 1 data using peak detection analysis of retention time window 0.30-14.50 min, with a mass range of 100 Da to 1200 Da. The extracted-ion chromatogram (XIC) window for data collection was 0.2 Da and apex peak tracking parameters were set to automatic with no smoothing. Data collection parameters consisted of an intensity threshold (counts) of 500 and a mass window of 0.02 Da with a retention time window of 0.20 min. A noise elimination level of 6 was applied and isotopes were removed. Peak heights for extracted ions were normalized against the total peak height of all extracted ions and standardized to a total ion count of 10 000. The results were exported in .csv format as a two dimensional data table in which rows and columns respectively represented analysed samples and the relative normalized peak heights of each detected mass spectrometric signal, i.e. as an Accurate Mass (m/z) and Retention Time (min) Pair (AMRTP).

### Data analysis

Temporal changes in BPI3V antibody titre were analysed using two-tailed paired *T*-test, and significant differences between treatment groups at sampling stages was assessed using two-tailed heteroscedastic *T*-test. SIMCA-P+ version 13.0 (Umetrics, Sweden) was used for multivariate metabolite marker selection. For SIMCA-P+ analysis, data was prefiltered to exclude AMRTPs with coefficient of variation greater than 50% in replicate quality control pools. All centroid data were Pareto scaled and analysed by unsupervised Principle Component Analysis (PCA) and supervised discriminatory analysis by Orthogonal Projections of Latent Structures-Discriminant Analysis (OPLS-DA). Unsupervised PCA models were generated at each sampling day to reveal potential relationships between treatment groups. Supervised analysis by OPLS-DA was performed to reveal potential markers of response to treatment in vaccinated calves compared to non-vaccinated calves at each sampling day. Robustness of final OPLS-DA discriminative models was assessed by setting a predictive model of each case in which 2/3 of the data (known treatment) was used to predict the remaining 1/3 (unknown treatment).

### Identification of potential metabolite markers

The elemental composition of selected parent compounds was determined in MassLynx using both positive and negative mode data. Mass uncertainty was set to 3mDa, odd and even electron state, carbon isotope filter of +/− 5% and elements included were C, H, O, N, P and S. Where applicable Na and K adduct elemental composition were determined with the respective element included in the analysis parameters. Elemental compositions were searched against PubChem and Chemspider online databases, and where possible Function 2 fragments were matched against Metlin, HMDB or Massbank databases. Where fragmentation spectra for the analyte in question was not available, in silico fragmentation was performed using Metfrag and Function 2 fragmentation data was validated against potential in silico fragments. Compounds revealed by database matching or in silico fragmentation had their identities confirmed against commercial analytical standards by UPLC-MS. Pooled plasma samples and individual standards (1 μM) were analysed under identical UPLC and mass spectrometric run conditions as utilized previously, and metabolite identities confirmed by matching retention time and Function 1 and Function 2 spectra (including low and high energy fragments and adducts).

## Results

### Haematological and immunological responses to vaccination

**Figure 2:**
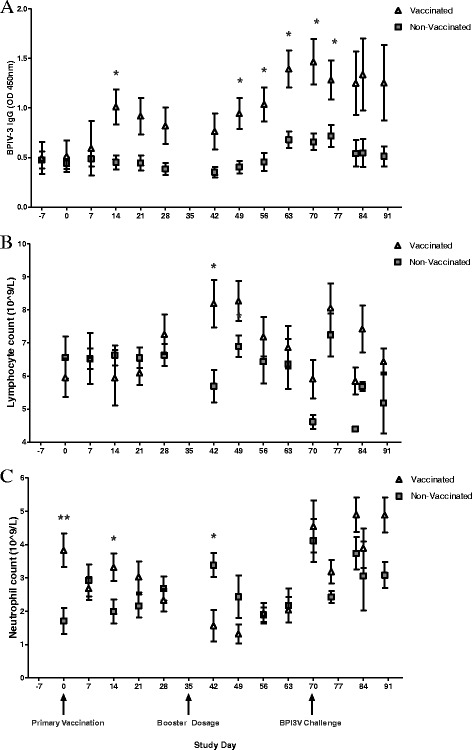
**BPI3V-IgG, Lymphocyte and Neutrophil variations in response to vaccination and subsequent BPI3V challenge. (A)** BPI3V antibody titre was determined in serum samples using Svanovir-PIV3 Ab Kit. **(B)** Lymphocyte and **(C)** neutrophil counts in blood. Significant differences in vaccinated animals compared to non-vaccinated animals at the same study day are indicated * *p* < 0.05, ** *p* < 0.01 and *** *p* < 0.001. Values represent mean ± S.E.M, *n* = 6.

Calves were monitored throughout the study to ensure normal health status and assess immunological responses to vaccination. Rectal temperatures taken at days 14, 28, 42 and 63 were within the reference parameters 38.3 to 39.4 °C with no significant differences observed between study groups (as illustrated in Additional file 1). Serum was obtained from all calves to determine weekly IgG anti BPI3V sera titres during vaccination procedures and to perform routine blood hematology analysis to validate calve health. The results of BPI3V IgG antibody titre screening are shown in Figure 2 in addition to lymphocyte and neutrophil counts obtained from hematology results. Prior to vaccination no significant differences in the BPI3V IgG antibody titre between study groups were observed (Figure 2A). IgG BPI3V antibody titres were significantly elevated (*p* < 0.05) in vaccinated calves compared to non-vaccinated calves at days 14, 49, 56 and 63. The BPI3V IgG antibody titre in vaccinated calves was shown to increase significantly (*p* < 0.05) from days 7 to 14 and significantly (*p* < 0.05) declined from day 14 to 28 whilst remaining significantly (*p* < 0.05) elevated compared to day 0. On administration of the second vaccine dosage (day 35) BPI3V IgG antibody titre increased (*p* < 0.05) from days 42 to 56, and remained significantly elevated in the vaccinated study group relative to non-vaccinated calves until day 63. These IgG responses indicate that the booster immunization was necessary to further increase the titres of BPI3V specific antibodies. Upon BPI3V challenge at day 70, BPI3V IgG antibody titre was maintained at significantly higher (*p* < 0.05) levels at days 70 and 75 in vaccinated calves compared to non-vaccinated calves indicating an adaptive primed immune response to infection. There was an observed drop in BPI3V IgG titre in vaccinated calves from day 70 to day 75 but this level was still significantly higher than that found in non-vaccinated calves (*p* < 0.05). There were no significant changes in the levels of BPI3V IgG titre in vaccinated or non-vaccinated calves throughout the remaining infection stages, with mean BPI3V IgG titres remaining higher in vaccinated calves compared to non-vaccinated.

The haematology results are illustrated in Additional file 1. Red Blood Cell count (RCC), White Blood Cell count (WCC), Hemoglobin content (HG), Mean Corpuscular Volume (MCV), Mean Corpuscular Haemoglobin (MCH), Packed Cell Volume (PCV) and Platelet count (PLT) measurements for all calves were within the specified reference parameters based on data from Brun-Hansen et al. [24] for calves at corresponding ages which are known to differ from adults. However, significant differences in lymphocyte and neutrophil count were observed between study groups at a number of points throughout the vaccination stages of the study whilst remaining within reference parameters for healthy animals. Figure 2B reveals significantly (*p* < 0.05) higher lymphocyte counts at day 42 (2 weeks post-booster immunization) in vaccinated animals compared to non-vaccinated. Furthermore, lymphocyte counts in vaccinated animals were found to rise significantly (*p* < 0.05) from day 14 to 42 and decreased from day 49 to 63, with no significant temporal changes observed in non-vaccinated animals. Figure 2C illustrates that neutrophil counts were significantly higher at day 0 (*p* < 0.01) and day 14 (*p* < 0.05) and significantly lower (*p* < 0.05) at day 42 in vaccinated compared to non-vaccinated animals. Neutrophil counts decreased significantly (*p* < 0.05) in vaccinated animals from day 14 to 42 and increased significantly (*p* < 0.05) in non-vaccinated animals from day 14 to 42. At post-BPI3V challenge stages, WCC, RCC and lymphocyte counts were outside reference parameters. There was a significant (*p* < 0.05) rise in WCC in non-vaccinated animals after BPI3V infection from days 70 to day 75 and WCC was higher than reference parameters from days 75 to 90 in vaccinated calves, and significantly (*p* < 0.05) higher at day 90 in vaccinated calves compared to non-vaccinated. RCC was elevated outside reference parameters throughout the study and was significantly (*p* < 0.05) higher in vaccinated calves at day 75 compared to non-vaccinated. Whilst No significant differences were observed in lymphocyte counts between study groups, lymphocyte counts were above the reference parameters in both study groups at day 75. There was a significant increase in lymphocyte counts in non-vaccinated animals from day 70 to 75 and a significant decrease from days 75 between days 82, with no significant variations observed in lymphocyte counts of vaccinated animals. There were no observed significant differences in neutrophil counts between study groups at corresponding sampling points post-infection, however, significant (*p* < 0.05) temporal variations were observed with a decrease from day 70 to 75 in non-vaccinated animals, and an increase from day 75 to 82 in both study groups. Despite being within the reference parameters, significant differences were observed in PCV at day 75, MCV at day 82 and MCH at day 75 between study groups.

### Selection of metabolomic markers of vaccine response via multivariate data analysis

**Figure 3:**
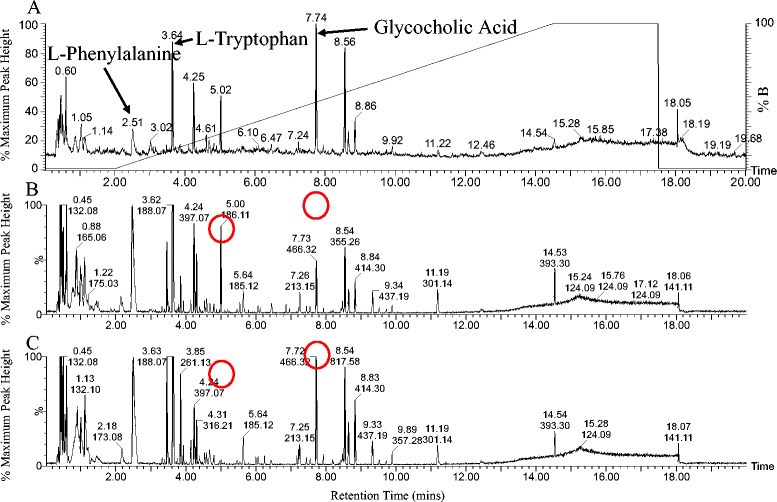
**Metabolomic profiling of bovine plasma by positive ionization UPLC-MS. (A)** Total Ion Chromatogram (TIC) of bovine plasma. Peaks relating to L-phenylalanine, L-tryptophan and glycocholic acid are indicated. The percentage of solvent B (99.9% acetonitrile, 0.1% formic acid) is indicated on right Y axis. **(B)** BPI chromatograms of plasma from a vaccinated calf at day 49 (two weeks after booster dosage), and **(C)** BPI chromatogram of plasma from a non-vaccinated calf at day 49. Representative differences between treatment groups are at the peaks indicated with retention times 5.00 and 7.73 min.

Representative Base Peak Intensity (BPI) chromatograms of plasma from both a vaccinated and non-vaccinated calf at day 49 are presented in Figure 3. When observing the acquired chromatograms there is obvious poor column retention of polar compounds between 1 min and 2 min. Between 2 min and 14.5 min chromatographic separation was excellent, with no observable overlapping peaks and minor differences between the acquired profiles of respective study groups (e.g. peaks at 5.00 min and 7.73 min) can be determined by visual examination. 3224 features were extracted from raw data using MarkerLynx and normalized to reduce the effects of batch to batch variation in subsequent analysis steps. In order to validate instrument performance landmark compounds in plasma (validated using pure standards) were used to analyse retention time deviation and mass accuracy between and within batch runs. The retention time deviation and mass accuracy of L-Phenylalanine, L-Tryptophan and Glycocholic Acid were determined in inter-run quality control pools and are presented in Additional file 2. Mass accuracy was consistent for all markers, with a maximum deviation of 2.26mDa. The maximum retention time deviation of peak top time was approximately 6 s. Maximum retention time deviation was observed with phenylalanine which can be accounted for by its wider elution time as illustrated in Figure 3. The excellent retention time stability and mass accuracy during and between separate analysis runs provides assurance in accuracy of the data collected.

**Figure 4:**
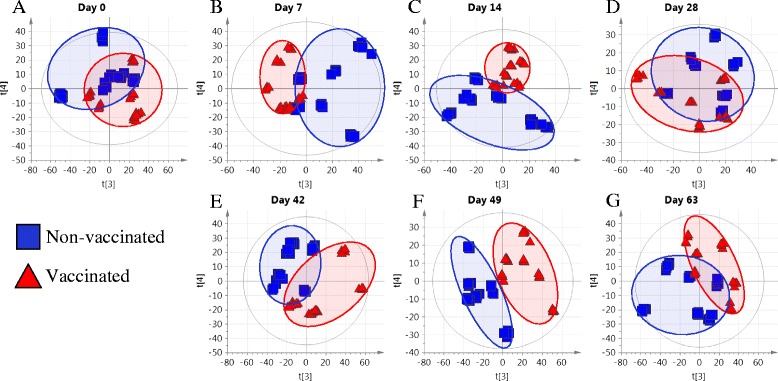
**Unsupervised PCA scores plot of UPLC-MS profiled plasma from vaccinated and non-vaccinated calves.** Unsupervised PCA scores plot of plasma from vaccinated and non-vaccinated calves at days 0, 7, 14, 28, 42, 49 and 63 are illustrated in Figures 4 **A-G** respectively.

Untargeted Principle Component Analysis (PCA) was performed in SIMCA-P+ v 13. The dataset was filtered to exclude variables with a coefficient of variation greater than 50% in replicate quality control pools. PCA models were constructed from 764 AMRTPs, and further divided into sub-models by sampling day. Replicate injections were tightly clustered on the PCA scores plot, indicating low inter-run variation in peak intensity. No injections were observed as having d-mox twice d-crit (possible outliers). The majority of variation explained by principle components (PC) 1 and 2 was due to variation between the three analysis runs (48.3 to 57.1%), and therefore for all stage PCA sub-models only PCs 3 and 4 were investigated. At days 7, 14, 42 and 49 there was separation on the PCA-scores plot between study groups when observing the variation in the data explained by principle components 3 and 4 shown in Figures 4B,C,E and F. Roughly 14 to 20% of the variation in the dataset was responsible for group separation in PCs 3 and 4 in substage PCA models. However, at days 28 and 63 there was a significant overlap between treatment groups (Figures 4D and G respectively). When the data from each analysis run was treated separately to remove run-to-run noise, group separation was achieved using principle components 1 and 2 only. Therefore, the only negative impact of combining data from separate analyses was that components representing analysis run separation could be ignored.

**Figure 5:**
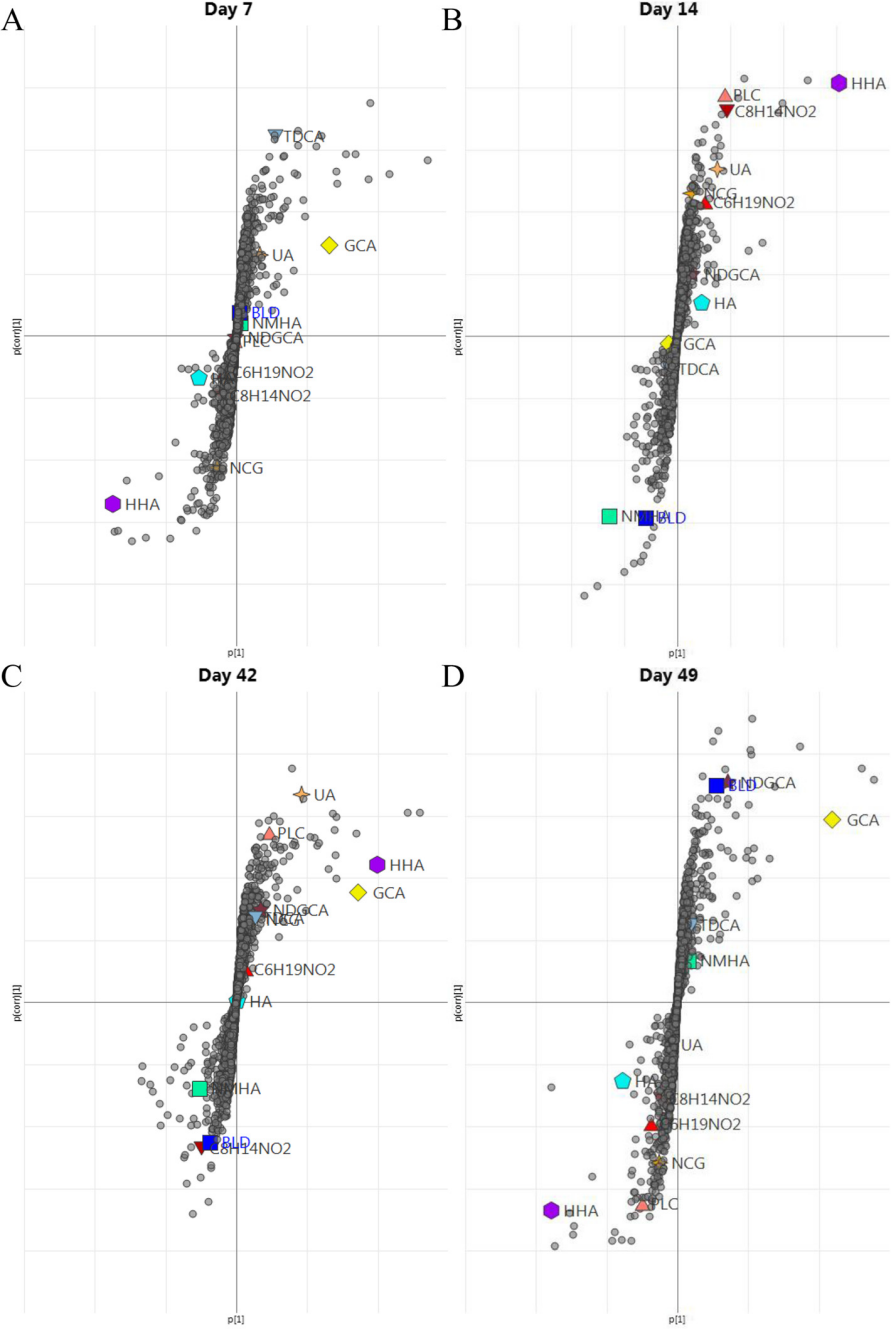
**S-plots of supervised OPLS-DA analysis of UPLC-MS profiled plasma from vaccinated and non-vaccinated calves.** Supervised OPLS-DA S-plot plots of plasma from vaccinated and non-vaccinated calves at days 7, 14, 42 and 49 are illustrated in Figures 5 **A-D** respectively. The location of AMRTPs on the S-Plot is a combination of influence (p [1]) and reliability (p(corr) [1]) on the discrimination of study groups by OPLS-DA. Identified vaccine response markers and AMRPTs selected throughout the study are marked on the S-Plot.

OPLS-DA was used for the selection of markers of immune response to vaccination. In order to validate OPLS-DA models, 2/3 of the randomly selected data (4 calves per group) was used to generate OPLS-DA models, with the remaining 1/3 used as a test set. The results from cross validation are illustrated in Additional file 3. At days 7, 14, 42 and 49 constructed models could accurately predict class allocation for the test set. At days 28 and 63 there was no correct classification for test samples, concurrent with the PCA scores plot in which there was no separation between study groups. Therefore, OPLS-DA models were constructed at days 7, 14, 42 and 49 for the selection of potential markers with all experimental calves used in the generation of these models. For all models the variables which contributed to study group discrimination in OPLS-DA models were selected on the criteria of a variable importance (V.I.P.) score > 1. The OPLS-DA S-plots of the metabolomic profiling of plasma from vaccinated and non-vaccinated calves at days 7, 14, 42 and 49 are illustrated in Figures 5A-D respectively. Many of the variables which contributed to the discrimination between study groups were related low energy fragments or adducts of parent compounds. 96 AMRTPs which demonstrated significant variation (*p* < 0.05, two-tailed heteroscedastic *T*-test) and a fold-change (FC) greater than 1.5 between treatment groups were selected (see Additional file 4). Significant AMRTPs were filtered to remove those with poorly consistent peak heights in replicate injections and high intergroup variability. Fragment ions and adducts were then identified by observing mass differences in co-eluting AMRTPS. Na and K adducts were observed as having mass differences of +22 and +38 respectively relative to the parent compound mass (M + H). Furthermore, by investigating function 2 fragmentation data, parent ions were selected as often having reduced peak intensity compared to Function 1.

### Identification of parent ions and AMRTPs

Final selection for parent ions was based on raw data, not normalized intensity, reducing the chance of including normalization induced false positives. AMRTPs with poorly consistent peak intensity in replicate infections were also removed. Out of the 16 final parent ions selected, 4 were removed as being artifacts of the normalization process. The 12 remaining AMRTP parent ions were selected for identification using a combination of database searching, spectral matching, elemental composition analysis and in silico fragmentation. For AMRTP identification function 2 MS/MS data was screened in Metlin for MS/MS fragmentation patterns. When available, if MS/MS fragmentation data was present in the database, MS/MS function 2 data in +ve or –ve ionization mode was screened for identification. Elemental composition of the 12 parent compound was determined in MassLynx. 6 of the 12 compounds chosen had % confidence fit of the isotope pattern between 90 and 100%. Function 2 fragmentation data was screened against the Metlin online database. The retention time and accurate mass of identified markers of immune response to vaccination are presented in Table 1 together with the percentage confidence of elemental composition determination in addition to potential database IDs.

**Table 1:** **Final AMRPTs selected as metabolomic markers of response to vaccination**

**AMRTP I.D.**	**Retention time (mins)**	**Measured mass (Da)**	**Elemental composition (M + H)**	**% Fit confidence**	**Mass Error (mDa)**	**PubChem I.D.**	**Compound**
0.4295_151.145	0.4295	151.145	C6H19N2O2	98.17	0.7		Unknown
0.4926_156.105	0.4926	156.105	C8H14NO2	99.66	1.4		Unknown
0.6158_169.039	0.6158	169.039	C5H5N4O3	99.48	−0.4	1175	Uric Acid (UA)^a^
1.4952_218.14	1.4952	218.14	C10H20NO4	95.95	−0.2	107738	Propionylcarnitine (PLC)
4.2427_180.066	4.2427	180.066	C9H10NO3	74.69	1.1	464	Hippuric Acid (HA) ^a^
4.5993_194.082	4.5993	194.082	C10H12NO3	54.33	−1.1	97479	N-Methylhippuric Acid (NMHA)
4.8821_184.097	4.8821	184.097	C9H14NO3	34.64	−0.2	149048	N-(Cyclohex-1-en-1-ylcarbonyl)glycine (NCG)
5.006_186.113	5.006	186.113	C9H16NO3	100	0.1	147412	Hexahydrohippuric Acid (HHA)^a^
7.1759_464.3	7.1759	464.3	C26H42NO6	96.92	−0.6	10253857	N-[(3α,5β,12α)-3,12-Dihydroxy-7,24-dioxocholan-24-yl]glycine (NDGCA)
7.7341_466.317	7.7341	466.317	C26H44NO6	16.22	−0.6	10140	Glycocholic Acid (GCA)^a^
8.2088_500.304	8.2088	500.304	C26H46NO6S	5.8	−0.8	44688	Taurodeoxycholic Acid (TDCA)^a^
8.5105_583.255	8.5105	583.255	C33H35N4O6	18.33	1.4	5280353	Biliverdin (BLD)^a^

Elemental composition of AMPRTs was calculated in MassLynx, with mass error of 3mDa, and elements C, H, O, P, S and N and % fit confidence for 3 isotopic peaks was calculated. Where possible spectral match score against function 2 data in Metlin is indicated. Compounds identified by pure standards are indicated with^a^.

**Figure 6:**
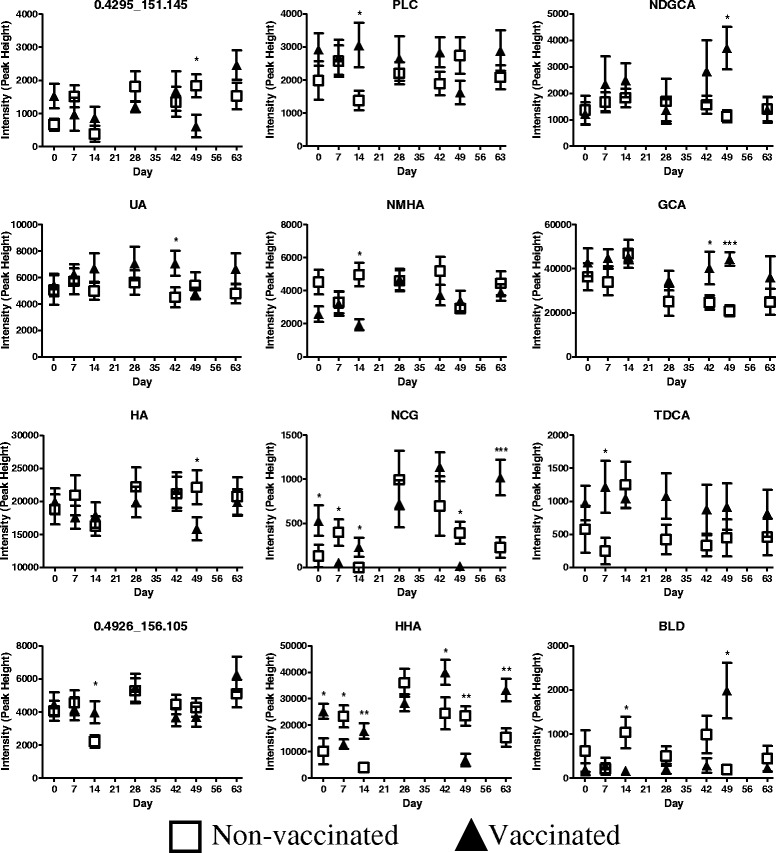
**Temporal changes in metabolite peak intensity throughout vaccination program.** Raw peak intensity (height) of the selected biomarkers at each sampling stage is indicated (*n* = 6). Significant changes in metabolite peak intensity between vaccinated and non-vaccinated calves at the corresponding study day (height) are indicated *P < 0.05, **p < 0.01, ***p < 0.001. Values represent mean ± S.E.M, *n* = 6.

Of the 12 potential markers identified to differ significantly between vaccination study groups, 5 had their identities confirmed using pure standards (Uric Acid (UA), Glycocholic Acid (GCA), Taurodeoxycholic Acid (TDCA), Biliverdin (BLD), Hippuric Acid, (HA) and Hexahydrohippuric Acid (HHA)). Putative identities based on PubChem database searching and in silico fragmentation using MetFrag were obtained for propionylcarnitine (PLC), N-[(3α,5β,12α)-3,12-dihydroxy-7,24-dioxocholan-24-yl]glycine (NDGCA), N-methylhippuric acid (NMHA) and N-(cyclohex-1-en-1-ylcarbonyl)glycine (NCG) - identities for the AMRTPs 0.4295_151.145 and 0.4926_156.105, could not be assigned. The influence (p[1]) and reliability (p(corr)[1]) of these markers on the OPLS-DA multivariate data analysis can be observed in the S-plots illustrated in Figures 5A-D. AMRTPs with high influence and reliability are indicated in the top right and bottom left corners of the S-plots. The variations in detected marker peak intensities between treatment groups throughout the study are indicated in Figure 6. These findings indicate that fluctuations in metabolite marker levels can occur throughout sampling stages due to environmental factors, as illustrated in the temporal variations apparent in non-vaccinated control animals. Elevated metabolite levels in vaccinated animals, as illustrated at day 14 for 0.4926_156.105, NCG, PLC and HHA, are therefore vaccine induced temporal increases in these markers which contrasts with the normal temporal decrease observed in non-vaccinated controls as a result of environmental factors. HHA and NCG were shown to differ significantly between study groups at day 0. Repeated measures ANOVA with bonferroni post-hoc test revealed significantly different temporal relationships between study groups day 0 to day 7 with HHA and NCG found to decrease significantly (*p* < 0.05) in vaccinated animals only from day 0 to day 7. Peak intensity of HHA and NCG differed significantly between treatment groups at a number of stages, with Pearson correlation showing significant positive correlation (*p* < 0.001, r = 0.982 and r = 0.951 in vaccinated and non-vaccinated animals respectively). Coupled with TDCA these were the only AMRTPs to differ at the earliest post vaccination sampling point (day 7), prior to observable difference in anti-BPI3V IgG titre between study groups. The significant up and down regulation of 0.4926_156.105 and NMHA respectively was limited to primary vaccine dosage, and is likely to be related to the adaptive immune response to vaccination (significant rise in IgG BPI3V antibody titre at day 14). NHMA was found to significantly decrease (*p* < 0.05) from day 0 levels in vaccinated animals at day 14. Significantly higher (*p* < 0.05) 0.4296_156.105 at day 14 in vaccinated animal was a result of vaccine induced deviation from the temporal decrease from day 7 to 14 observed in non-vaccinated control animals. The remaining identified markers were found to differ significantly during the phase of secondary immune response. Peak intensity of UA was significantly higher (*p* < 0.05) in vaccinated calves compared to non-vaccinated calves at day 42 and was significantly higher (*p* < 0.05) than day 0 levels in vaccinated animals at days 7, 14 and 28. GCA was significantly up-regulated in vaccinated calves at days 42 and 49, however, this was a result of booster induced deviation in vaccinated animals which differed from the temporal profile observed in control non-vaccinated animals. Peak intensities of 0.4295_151.145 and HA were found to be significantly lower in vaccinated calves at day 49 and significantly lower (*p* < 0.05 and *p* < 0.01 respectively) than day 0 levels in vaccinated animals. BLD and NDGCA were up-regulated at day 49 in vaccinated animals, significantly higher (*p* < 0.05) than day 0 levels. Furthermore, HHA and NCG levels were found to be significantly correlated with HA throughout the study in vaccinated and non-vaccinated animals (HHA and HA, *p* < 0.001, r = 0.971 in vaccinated animals and *p* < 0.01 and r = 0.894 in non-vaccinated animals; NCG and HA, *p* < 0.01, r = 0.917 in vaccinated animals and *p* < 0.05, r = 0.761 in non-vaccinated animals).

## Discussion

Vaccine development strategies have typically focused on the induction of specific antibody responses to antigenic molecules as an indicator of effective immune stimulation (whole virus or viral proteins) with little understanding of how antigens elicit particular immune responses [6]. Whilst this approach has resulted in the majority of vaccines available today, new vaccine development is increasingly costly and it is imperative that the least promising candidates are eliminated from investigation as early as possible. Whilst the identification of immune correlations, in particular proteins such as interleukins and cytokines, has helped to improve the understanding of vaccine mediated immune response mechanisms, emerging metabolomic techniques may offer a novel perspective to vaccine development and selection. This is possible as metabolites are the ultimate end stage products or mediators of biological processes and can provide a holistic view of underlying physiological processes. This study has for the first time employed an untargeted UPLC-MS metabolomic profiling approach to identify plasma components altered by primary and secondary immune responses to an intranasal vaccination containing live modified virus. Vaccination with Pfizer Rispoval® PI3 + RSV resulted in an increase of BPI3V specific IgG antibody titre in vaccinated calves. The IgG responses indicated that the booster immunization was necessary to further increase the titres of BPI3V antibodies. This stimulated IgG response is characteristic of the immune responses typically seen in two dosage vaccination experiments. Furthermore, the high IgG titre maintained in vaccinated animals following experimental challenge with BPI3V indicates a primed immune response to infection. Conversely the non-vaccinated animals showed no significant alterations in BPI3V specific IgG titre during vaccination stages but at post-BPI3V challenge displayed a rise in WCC indicating a stimulated immune response in the absence of prior infection. We can therefore characterize the markers identified in this study which showed altered abundance between vaccinated and non-vaccinated study groups as being related to the immunological responses involved in primary and secondary immune-stimulation. Metabolomic profiling initially revealed a panel of 96 features which differed significantly between calves vaccinated intranasally with Pfizer Rispoval® PI3 + RSV and non-vaccinated calves throughout the study. These features were further refined to identify 12 high confidence parent compounds, 10 of which were identified based on database searching and spectral matching, with 6 further validated using pure standards. Based on the observed stimulated IgG response following two intranasal vaccine dosages (characteristic of the immune response typically seen in two-stage dosage vaccine programs [6]), these markers can be characterized as being related to the responses involved in primary and secondary immune stimulation. All detected markers were shown to be present in plasma prior to vaccine administration, therefore their altered abundance reflects the processes of stimulation of the mucosal immune system following exposure of animals to modified live viral vaccine, and are not associated with vaccine breakdown products.

A significant increase in BLD (10.26 FC) plasma peak intensity in vaccinated calves compared to non-vaccinated calves was observed two weeks after the second Pfizer Rispoval® PI3 + RSV dosage (day 49). BLD is degraded from heme groups by Heme Oxygenases (HO) [25] and rapidly reduced to Bilirubin by Biliverdin Reductase [26]. BLD and the HO isoform HO-1 are involved in promoting anti-inflammatory responses. BLD suppresses IL-2 production and T cell proliferation [27], and activates the aryl hydrocarbon receptor (AHR) [28] regulating T helper cell phenotype towards an anti-inflammatory regulatory T cell, or a pro-inflammatory T helper 17 effector function [29,30]. HO-1, produced from circulating monocytes during acute inflammatory states, has immunomodulatory function by supporting the proliferative capacity and activation of CD4(+) and CD8(+) T cells via CD14(+) monocytes, preventing DC maturation [31-33] and controlling Th1 pro-inflammatory cytokine production [34,35]. The observed increased plasma levels of BLD may be due to either increased HO-1 activity at a rate which cannot be matched by Biliverdin Reductase, or an unknown modulatory effect on Biliverdin Reductase affecting the rate at which it reduces BLD to bilirubin **-** strongly supporting BLD and HO-1 involvement in the promotion of anti-inflammatory responses as the immune stimulation response following vaccine dosage is deactivated to normal status.

Plasma peak intensities of a number of conjugated Bile Acids (BAs) were found to differ significantly between vaccinated and non-vaccinated calves, with increased TDCA (4.87 FC) after primary vaccine dosage (day 7, prior to observable differences in IgG titre) and increased GCA (1.63 and 2.12 FC) and NDGCA (1.80 and 3.29 FC) after secondary dosage (day 42 and 49 respectively) in vaccinated animals. The up-regulation of different BA conjugates at different stages following vaccination suggests that Cholic acid conjugation may allow for mediation of different immune response pathways, dependent on the amino acid that is conjugated. To date the only other published study assessing metabolomic responses to vaccination reported an up-regulation of serum cholesterol levels at days 5 and 10 in rabbits after primary and secondary immunization (using human red blood cells) respectively [1]. As cholesterol is a precursor to Cholic acid, and hence GCA and TDCA, it’s up-regulation could be due to an increased demand for immuno-regulatory BAs. BAs have recently been demonstrated to function as signaling molecules with immunomodulatory effects resulting from their activation of the nuclear receptor Farsenoid X Receptor (FXR) [36,37] and the G-protein coupled receptors Transmembrane G protein coupled receptor 5 (TGR5) [38,39] and the Formyl-peptide receptors (FPR) [40]. TGR5, present in a variety of cells of the immune system including alveolar macrophages and dendritic cells [38,41], can be activated by BAs to stimulate cAMP production which inhibits pro-inflammatory cytokines secretion (TNF-α, IL-1β, IL-1α, IL-6 and IL-8) [38]. This activation favours more hydrophobic BAs [38], with TDCA more likely to activate TGR5 in vivo compared to GCA or NDGCA and its’ up-regulation at day 7 in vaccinated calves illustrates a potential role in arresting innate inflammatory response in favour of the progression of adaptive immunity through the activation of TGR5. Dendritic cells demonstrate FXR modulated function following BA treatment, resulting in increased production of IFN-γ producing T-cells and reduced airway eosinophilia and macrophage influx after OVA airway stimulation [42]. Activation of FXR by synthetic bile acid ligands also results in NK T-cell inhibition and reduced pro-inflammatory osteopontin production which indicates FXRs role in maintaining immune homeostasis [43]. Both TDCA and GCA have been shown to activate FXR in vivo, upon transportation into FXR expressing cells [44]. However, the consequences of BA driven FXR modulation would favour an adaptive immune response, which would therefore be associated with secondary booster dosage stages where increased plasma levels of GCA and NDGCA were observed in vaccinated animals. Significant up-regulation of circulating plasma levels of GCA could provide a method by which pro-inflammatory responses at the site of infection are limited after secondary exposure to viral infections. GCA induced activation of FXR at post-booster stages in vaccinated animals may reduce macrophage and neutrophil influx to the site of infection and increase the levels of INF-γ producing CD4+ and CD8+ T-cells, promoting an adaptive immune response.

Significantly higher levels of PLC (2.21 FC) in vaccinated relative to non-vaccinated calves were observed at day 14 post initial vaccine dosage, suggesting a potential role of PLC in either mediating adaptive immune responses following primary immune stimulation, or as an end-stage metabolite produced as a result of these processes. PLC has been demonstrated to modulate innate immune response following transplantation by preventing neutrophil, CD4+, CD8+, ED1+ and MHCII cell infiltration upon transplantation, protecting grafts from oxidative stress injury [45]. In vivo PLC treatment resulted in significant decrease in the release of leukocyte adhesion molecules into plasma (E-selectin, P-selectin, L-selectin ICAM-1 and VCAM-1) [46] suggesting PLC’s action in modulating leukocyte function and trafficking, with a potential role in reducing immune cell recruitment to sites of active viral replication with the progression of adaptive immunity associated with increased BPI3V IgG production at day 14 in this study. The immunomodulatory effects of PLC are further demonstrated by its’ potential to reduce platelet activating factors when exposed to neutrophils in vitro and in vivo [47]. The significant up-regulation of PLC at day 14 may therefore be due to its’ anti-inflammatory effect in arresting pro-inflammatory leukocyte recruitment in favour of adaptive immune response.

UA released from injured cells activates the NALP3 inflammasome leading to IL-1β production [48], but the specific mechanism by which UA promotes its immunosuppressive effects is not fully understood [49,50]. Significantly higher levels of UA (1.57 FC) were observed in vaccinated relative to non-vaccinated calves at day 42 (1 week post second vaccine dosage) and would suggest an increased population of cytotoxic T cells responding to re-exposure to the live attenuated vaccine. Whilst there has been no previously reported evidence of the direct immunosuppressive properties of HA or HHA, significantly lower levels of HA (21.70 FC) were observed in vaccinated animals at day 49 (2 weeks post booster immunization), and significant differences in the plasma peak intensity of HHA in vaccinated animals compared to non-vaccinated at days 7 (−1.82 FC), 14 (4.45 FC) post initial immunization and days 42 (1.63 FC), 49 (− 3.41 FC) and 63 (2.17 FC), 1, 2 and 4 weeks post booster immunization respectively. Reduced plasma HA levels in vaccinated calves compared to non-vaccinated calves, suggests that it does not exert a direct anti-inflammatory effect. HA may instead be involved in the mediation of immune response through its’ metabolism to produce compounds which exert a direct immuno-modulatory effect. Hippuric acid is an acyl-glycine, formed through the action of N-acyltransferase involving Acyl-CoA. Circulating HA in mammals is primarily derived from the metabolism and glycine conjugation of phenolic dietary constituents [51]. The metabolites HHA, NMHA and NCG have acyl-glycine like fragmentation patterns, with HHA and NCG levels found to significantly correlate with HA. As dietary composition was consistent between study groups and significant variation in HA occurred only at post-booster stages, the reduced plasma HA levels may result from reduced N-acyltransferase activity (due to increased metabolic demand for acyl-CoA) coinciding with a marked reduction in plasma levels of other metabolites (HHA and NCG).

In conclusion, this study has demonstrated the ability of untargeted UPLC-MS metabolomics to differentiate between vaccinated and non-vaccinated animals via the profiling of metabolite constituents in plasma. A number of plasma constituents were found to be associated with initial and/or booster vaccine dosage events and were therefore characterized as corresponding to primary and/or secondary immune stimulation. The markers associated with primary immune stimulation to vaccine administration, particularly TDCA and HHA which varied in plasma prior to observable variations in BPI3V IgG, may find application as early diagnostic markers to screen for immunogenic candidates during vaccine selection investigations, particularly for vaccines targeting routes of pathogen entry such as through the respiratory tract. The metabolites GCA, NDGCA, UA and BLD, which were altered within plasma at the secondary vaccine dosage stages, may additionally represent key diagnostic markers of established immune protection in animals. A number of the selected markers have demonstrated immunological properties, affecting key cell signaling pathways and receptors located on cells of the immune system. Further validation studies are required to expand upon this initial proof of concept study to discern whether the differences observed in these metabolite marker levels as a result of vaccination are repeatable in a larger external sample set comprising animals of varying ages, genders and breeds coupled with more frequent sampling. However, this study’s findings illustrate the potential for untargeted metabolomics to identify key metabolite immune correlates which relate to specific cell signaling pathways involved in immune responses. The assessment of such metabolite markers may provide greater information on the immune pathways stimulated (compared to traditional IgG or IgA serology) by vaccine formulations, and in particular benchmarking early metabolomic responses to highly immunogenic vaccine formulations could provide a means of rapidly assessing new vaccine candidates. Furthermore, a greater understanding of the metabolite pathways altered as a result of vaccination could allow for targeted vaccine design (antigens, adjuvants and nanoparticle carrier systems) to stimulate key immunogenic pathways which can be assessed at the metabolite level. Currently, metabolomics does not lend itself easily to routine vaccine candidate screening, due to the requirement of costly analytical equipment and skilled operators. However, recent advances in portable mass-spectrometers, such as the 908 Devices handheld system (908 Devices, Boston, USA), or the more user friendly Microsaic 4000 MiD® (Microsaic Systems, Surrey, UK), may eventually see the use of metabolites as routine markers for the evaluation of vaccine candidate immune function and immunogenicity. Future research will seek to expand upon the physiological role of these metabolomic markers and focus on establishing the biological pathways in which they may play a role in immuno-modulatory activities.

## Additional files

Additional file 1:
**Rectal temperatures and haematology findings throughout the study.** Routine blood haematology was performed on serum samples from vaccinated and non-vaccinated study groups at days 0, 7, 14, 21, 28, 42, 49, 56, 63, 70, 75, 82, 84 and 90 post-initial immunization. Analysis of rectal temperature was performed at days 14, 28, 42 and 63. Values represent mean ± S.E.M. Additional file 2:
**Inter-run quality control parameters for UPLC-MS.** Retention time deviation and mass accuracy of validated peaks corresponding to L-phenylalanine, L-tryptophan and glycocholic acid analysed in inter-run quality control pools. Additional file 3:
**Cross-validation of supervised OPLS-DA analysis.** OPLS-DA fitting and predictive statistics generated from cross-validation using 2/3 randomly selected dataset for the generation of predictive models and the remaining 1/3 as a test set. Additional file 4:
**Selected AMRTPs of plasma metabolomic markers of response to vaccination.** Combined list of markers selected using OPLS-DA at days 7, 14, 42 and 49 and filtered to exclude those with *p* < 0.05, FC < 1.5 and V.I.P. score < 1.

## Abbreviations

BRD: Bovine respiratory disease
BPI3V: Bovine Parainfluenza Type3 Virus
UPLC-MS: Ultra Performance liquid chromatography-mass spectrometry
AMRTP: Accurate mass retention time pair
PCAk: Principle component analysis
OPLS-DA: Orthogonal projections to latent structures-discriminant analysis
PC: Principle component
MS/MS: Tandem mass spectrometry
UA: Uric acid
GCA: Glycocholic acid
TDCA: Taurodeoxycholic acid
BLD: Biliverdin
HA: Hippuric acid
HHA: Hexahydrohippuric acid
PLC: Propionylcarnitine
NMHA: N- N-Methylhippuric acid
NCG: N-(cyclohex-1-en-1-ylcarbonyl)glycine
FXR: Farsenoid X Receptor
TGR5: Transmembrane G-Protein Coupled Receptor 5
BA: Bile Acid
NALP3: NACHT, LRR and PYD domains-containing protein 3
